# Gross primary production of global forest ecosystems has been overestimated

**DOI:** 10.1038/srep10820

**Published:** 2015-06-01

**Authors:** Jianyong Ma, Xiaodong Yan, Wenjie Dong, Jieming Chou

**Affiliations:** 1 State Key Laboratory of Earth Surface Processes and Resource Ecology, Beijing Normal University, Beijing 100875, China

## Abstract

Coverage rate, a critical variable for gridded forest area, has been neglected by previous studies in estimating the annual gross primary production (GPP) of global forest ecosystems. In this study, we investigated to what extent the coverage rate could impact forest GPP estimates from 1982 to 2011. Here we show that the traditional calculation without considering the coverage rate globally overestimated the forest gross carbon dioxide uptake by approximately 8.7%, with a value of 5.12 ± 0.23 Pg C yr^−1^, which is equivalent to 48% of the annual emissions from anthropogenic activities in 2012. Actually, the global annual GPP of forest ecosystems is approximately 53.71 ± 4.83 Pg C yr^−1^ for the past 30 years by taking the coverage rate into account. Accordingly, we argue that forest annual GPP calculated by previous studies has been overestimated due to the exaggerated forest area, and therefore, coverage rate may be a required factor to further quantify the global carbon cycle.

Vegetation gross primary production (GPP) is the largest CO_2_ flux of the carbon cycle in terrestrial ecosystems and impacts all of the carbon cycle variables^1^. Forest ecosystems, as important components of the terrestrial carbon cycle, always store much larger amounts of carbon than other terrestrial ecosystems^2^. Therefore, accurate quantification of GPP in forest ecosystems on a global scale would provide scientific foundations for predicting future changes in atmospheric carbon dioxide and climate, and for defining management options for the global carbon cycle^3^.

Quantifying global-scale GPP has been difficult because there are no direct measures at scales greater than the leaf level^4^. Currently, the traditional methods for estimating spatial-temporal variations in forest ecosystem production mainly include existing forest inventory, satellite remote sensing databases, inverse modeling using atmospheric transport models and atmospheric CO_2_ observations, and process-based carbon cycle model simulations. Because of the structural discrepancies^5^,^6^, estimates differ substantially in terms of magnitude and spatial distribution on regional and global scales using different methods^6^,^7^. In general, the uncertainty caused by method structural differences is inevitable during the estimation process. Note, however, that other external factors which may potentially impact the estimations cannot be neglected regardless of method. For example, forest coverage rate (%), a critical variable for gridded forest area, is always neglected by calculations related to global annual carbon fluxes. In previous GPP research, for the gridded forest area, it was simply assumed that each grid was 100% covered by a given forest type, rather than taking the realistic coverage rate into consideration^1^,^8^,^9^. This inappropriate hypothesis would directly exaggerate the forest area and therefore overestimate the global annual GPP of forest ecosystems to a certain extent; in addition, it is true that some grids not categorized as forest on remote sensing land cover maps might also have a certain amount of forest area, however, these parts of GPP was completely not included in previous studies due to directly neglecting the lower forest coverage rate. Similarly, this kind of hypothesis would diminish the forest area to a certain degree and therefore underestimate the annual GPP of forest ecosystems.

In this study, based on forest ecosystem carbon budget model for china (FORCCHN), we reevaluated the global annual GPP (Pg C yr^−1^) of forest ecosystems by incorporating forest coverage rate of forest grids (Fig. 1b) and non-forest grids (Fig. 1c) into calculation, and further investigated the extent to which forest coverage rate could impact the global annual GPP at a grid resolution of 0.5° × 0.5° over the period of 1982−2011.

## Results

We simply compared the global GPP(g C m^−2^ yr^−1^) generated by FORCCHN with other studies related to seven light use efficiency(LUE) models’ outputs of five forest types during 2000−2010 (ref. 9) and seven estimations of forest ecosystem during 1993−2006 (ref. 1). Our model yielded comparable GPP per unit area as other methods on a global scale(Fig. 2). For example, FORCCHN generated a GPP of 841.4 and 2748.7 g C m^−2^ yr^−1^ for evergreen needleleaf forest (ENF) and evergreen broadleaf forest (EBF), which is ~12% and ~11% higher, respectively, than the average value of the seven LUE models (750.4 and 2479.1 g C m^−2^ yr^−1^). Meanwhile, the smallest discrepancy between our result and ref. 9 occurred in mixed forest (MIF) with GPP values of 1140.4 and 1144.3 g C m^−2^ yr^−1^, respectively, and a corresponding difference of only −3.9 g C m^−2^ yr^−1^. In terms of forest ecosystem GPP, there were no particularly obvious discrepancies among the seven estimations, with a range varying from 1156.7 g C m^−2^ yr^−1^ for the LUE method to 1484.7 g C m^−2^ yr^−1^ for the MIAMI method. Meanwhile, the GPP generated by FORCCHN for the period of 1993−2006 was 1329.1 g C m^−2^ yr^−1^, which is much closer to the average value of the seven estimations (1305.4 g C m^−2^ yr^−1^).

**Figure 1:**
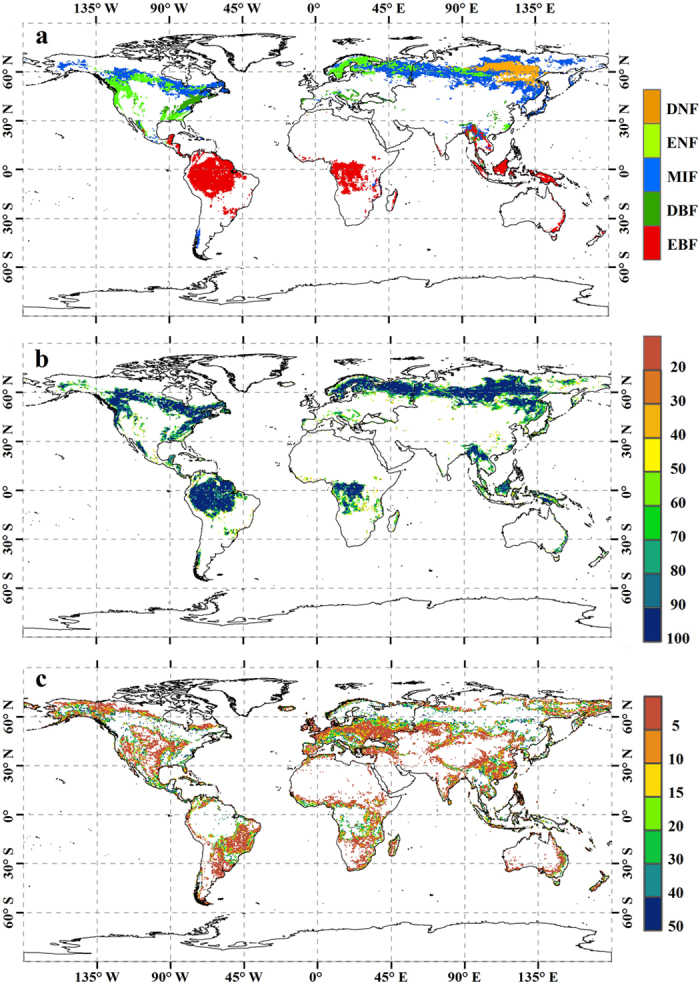
Global spatial distribution of forest at a grid resolution of 0.5° × 0.5°. **a**, IGBP-DIS forest classification. **b**,Forest coverage rate of forest grids(%).**c**,Forest coverage rate of non-forest grids(%).DNF, ENF, MIF, DBF and EBF represent deciduous needleleaf forest, evergreen needleleaf forest, mixed forest, deciduous broadleaf forest and evergreen broadleaf forest, respectively.The figure was produced using ArcGIS 10.1.

To further investigate the rationality of applying the FORCCHN model to evaluate the GPP per unit area of forest ecosystem on a global scale, a comparison concerning the global spatial distributions of averaged GPP (g C m^−2^ yr^−1^) derived from FORCCHN and ref. 8 over the 1982−2011 period is shown in Fig. 3. Here, we extracted and recalculated the 30-year averaged GPP from ref. 8 at a resolution of 0.5° latitude by 0.5° longitude to match the forest distribution of IGBP-DIS classification (Fig. 1a). Fig. 3 shows that both of the spatial distributions of GPP are similar with regard to the largest values occurring in the equatorial tropics followed by monsoonal subtropical regions (e.g. south and east Asia), and humid temperate regions in eastern North America and western Europe. Boreal forests show a clear longitudinal gradient in northern Eurasia where GPP in boreal zone decreases toward the east, where the main forest type is deciduous needleleaf forest (DNF). Note, however, that GPP derived from FORCCHN in tropical rain forest (20 °S−20 °N) was generally ~300 g C m^−2^ yr^−1^ smaller than that of ref. 8, except for parts of Africa. Conversely, compared with FORCCHN outputs, ref. 8 captured an obviously lower estimate in south-central Africa with a difference of approximately −900 g C m^−2^ yr^−1^; presumably in response to the reclassification of tropical savanna into C3, C4 and C3/C4 types in ref. 8.

As analyzed above, our model generated comparable GPP per unit area as other related studies on a global scale; thus, it is convincing enough to detect the impacts of coverage rate on global annual GPP of forest ecosystems using the FORCCHN model. Here, we computed two GPP results based on two circumstances of forest coverage rate. Calculation I, termed ‘100% coverage’, is a traditional assumption that each grid is 100% covered by a given forest type and is generally adopted by most carbon flux studies at regional or global levels. Calculation II, termed ‘realistic coverage’, is a new strategy that takes the IGBP-DIS coverage rate into account to reflect the actual forest area of each grid. Because some land cover grids not categorized as forest might also have a certain amount of forest area, both realistic forest coverage of forest grids (Fig. 1b, 4) and non-forest grids (Figs. 1c,4) are taken into account in Calculation II.

The global spatial comparsion of annual GPP (Tg C yr^−1^ grid^−1^) derived from two calculations during 1982−2011 is given in Fig. 5.It is evident that the difference between ‘100% coverage’ and ‘realistic coverage’ for forest grids is similar with the spatial distribution of IGBP-DIS coverage rate to some extent. For example, the overestimated GPP in most forest varies within the minimum range of 0−0.5 Tg C yr^−1^ grid^−1^ (Fig. 5d) when the corresponding coverage rate is more than 90% (Fig. 1b). Because of the lower coverage rate (40%−70%) along the periphery of EBF between 20 °S and 20 °N (Fig. 1b), the largest overestimation of GPP is found with a value of 3−6 Tg C yr^−1^ grid^−1^ (Fig. 5d). Note, however, that the ‘100% coverage’ does not always overestimate annual GPP at each forest grid compared with the ‘realistic coverage’. It is particularly true for some DNF and ENF girds in the Northern Hemisphere (Fig. 5d), where the difference between two calculations has been inversely underestimated by 0−0.1 Tg C yr^−1^ grid^−1^. This phenomenon can be attributed almost entirely to the fact that each grid of DNF and ENF consists of ~14% and ~15% MIF, respectively (Fig. 4). The relatively higher GPP per unit area of MIF might counteract the effects of non-forest coverage, and increase total GPP of these DNF and ENF grids. With respect to the difference between ‘100% coverage’ and ‘realistic coverage’ for non-forest grids, the underestimated GPP in most grids varies within the range of 0−0.5 Tg C yr^−1^ grid^−1^ (Fig. 5c,d) when the corresponding forest coverage is less than 5% (Fig. 1c). Meanwhile, due to the higher forest coverage (20%−50%) along the periphery of forest ecosystems (Fig. 1c), the largest underestimation of GPP is observed with a value of 1.2−4.5 Tg C yr^−1^ grid^−1^ (Fig. 5c,d).

The long-term changes in GPP estimates are consistent between the two calculations. Both calculations show a significant GPP increase (p < 0.001) from 1982 to 2011 regardless of forest type (Fig. 6), which is broadly consistent with the trend of terrestrial vegetation productivity given by 18 CMIP5 earth system models over the 1986−2005 period^10^. In terms of averaged annual GPP in the ‘100% coverage’ calculation, the forest types with the greatest carbon dioxide uptake are EBF, MIF, and ENF, with annual GPP of 39.58 ± 3.49 (mean ± 1 standard deviation), 10.84 ± 0.96 and 4.25 ± 0.72 Pg C yr^−1^, respectively (Table 1). Moreover, the global GPP estimates of forest ecosystems is 58.83 ± 5.61 Pg C yr^−1^, which is comparable to the observation-based estimations of 52.61−67.54 Pg C yr^−1^(ref. 1) and satellite-based simulations of 37.59−59.77 Pg C yr^−1^(ref. 9). As far as ‘realistic coverage’ calculation is concerned, the corresponding annual GPP estimates in EBF, MIF and DNF decrease to 35.13 ± 3.5, 9.65 ± 1.02 and 1.23 ± 0.13 Pg C yr^−1^, respectively, while ENF and deciduous broadleaf forest (DBF) inversely increase to 4.51 ± 0.75 and 3.19 ± 0.47 Pg C yr^−1^, and gross carbon dioxide uptake by global forest ecosystems is reduced with an annual GPP of 53.71 ± 4.83 Pg C yr^−1^, which is comprised of 46.19 ± 4.24 Pg C yr^−1^ for forest grids and 7.52 ± 1.59 Pg C yr^−1^ for non-forest grids(Table 1). Overall, compared with the ‘realistic coverage’, the ‘100% coverage’ calculation, which was generally adopted by most previous studies, has overestimated annual GPP across all forest types with exception of ENF and DBF. In terms of global forest ecosystems, the annual GPP is approximately overestimated by 5.12 ± 0.23 Pg C yr^−1^, accounting for ~8.7% of the global GPP estimates in the ‘100% coverage’ calculation.

## Discussion

**Figure 2:**
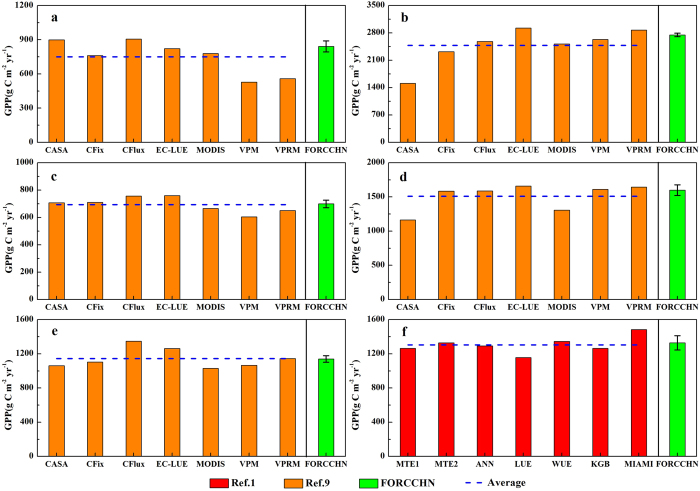
Comparison GPP per unit area (g C m^−2^ yr^−1^) derived from FORCCHN with other related researches for globally different forest types. **a**, ENF. **b**, EBF. **c**, DNF. **d**, DBF. **e**, MIF. **f**, forest ecosystems. Orange, red, and green histograms represent the multi-year averaged GPP from ref. 9, ref. 1, and FORCCHN, respectively. Error bars refer to one standard deviation. Ref. 9 consists of seven light use efficiency(LUE) models’ outputs for five forest types over the period of 2000−2010: Carnegie–Ames–Stanford Approach (CASA); Carbon Fixation (CFix); Carbon Flux (CFlux); Eddy Covariance-Light Use Efficiency (EC-LUE); Moderate Resolution Imaging Spectroradiometer (MODIS) GPP algorithm; Vegetation Photosynthesis Model (VPM) and Vegetation Photosynthesis and Respiration Model (VPRM). Ref. 1 is composed of seven estimations for forest ecosystems over the period of 1993−2006: Model Tree Ensemble 1 (MTE1); Model Tree Ensemble 2 (MTE2); Artificial Neural Network (ANN); Light Use Efficiency (LUE); Water Use Efficiency (WUE); Köppen-Geiger cross Biome (KGB) and MIAMI Model (MIAMI). The figure was produced using OriginPro 8.1.

**Figure 3:**
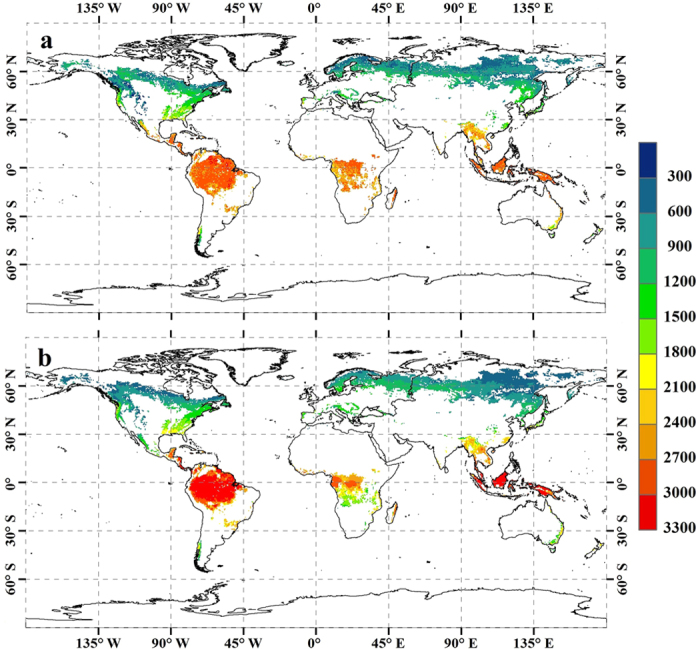
A spatial comparison of global forest averaged annual GPP (g C m^−2^ yr^−1^) during 1982−2011. a, FORCCHN. b, Ref. 8. The figure was produced using ArcGIS 10.1.

At present, different methods used to quantify the global annual GPP (Pg C yr^−1^) of forest ecosystems show substantial disagreement. These uncertainties are suggested to arise from the discrepancies in GPP per unit area (g C m^−2^ yr^−1^) and forest area. In terms of GPP per unit area, significant uncertainties related to the climate-driven ecosystem models are due to differing model sensitivities to climate parameters and uncertain global climatological datasets^11^,^12^; in addition, uncertainties with the remote sensing driven^13^ or flux-tower based^1^ semi-empirical models are due to the fact that GPP is simulated as a function of leaf area index (LAI) and fraction of absorbed photosynthetically active radiation (fPAR) or greenness indices such as the normalized difference vegetation index (NDVI)^14^, which are often contaminated by atmospheric interference, and may result in a misleading conclusion when vegetation becomes stressed^15^. As discussed above, moderate discrepancies between our results and other studies, shown in Fig. 2 and Fig. 3, are reasonable and inevitable because of the method structural differences. This is also the reason why we concluded that FORCCHN can yield comparable GPP per unit area (g C m^−2^ yr^−1^) as other methods on a global scale. What is more, there are some literatures showing that nitrogen(N) deposition has an impact on the carbon dioxide uptake by temperate, boreal and subtropical forest ecosystems in the Northern Hemisphere^3^,^16^, and Luyssaert *et al.* also proved that not accounting for N deposition resulted in a mean 11% lower net primary production (NPP) across European forests by comparing the BIOME-BGC simulations with and without N cycle^17^.Accordingly, N deposition not taken into account by FORCCHN model in present study might lead to underestimation of GPP to a certain degree.

**Figure 4:**
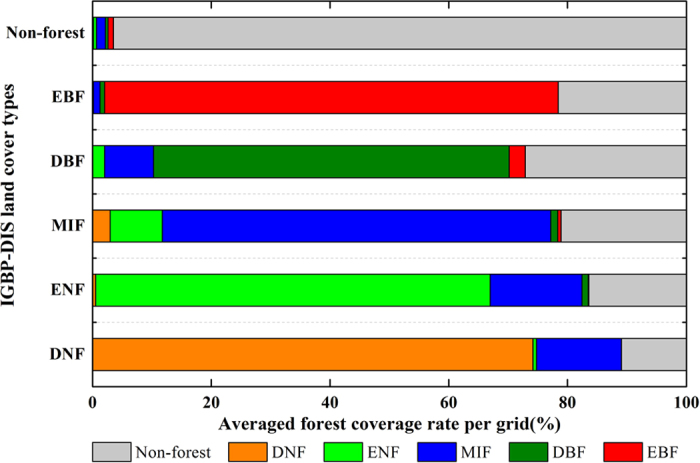
Averaged forest coverage rate(%) of the given IGBP-DIS land cover types at each grid. The figure was produced using OriginPro 8.1.

**Figure 5:**
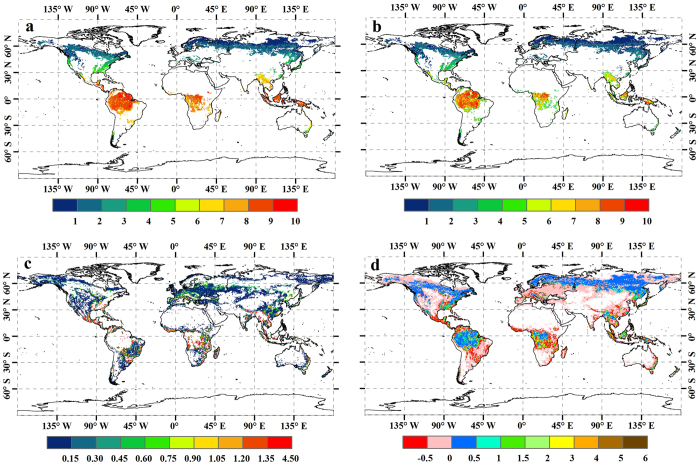
A spatial comparison of global forest annual GPP (Tg C yr^−1^ grid^−1^) during 1982−2011 under different coverage rate. **a**,100% coverage. **b**,Realistic coverage of forest grids. **c**, Realistic coverage of non-forest grids.**d**, Difference between **a** and **(b + c)**. The figure was produced using ArcGIS 10.1.

**Figure 6:**
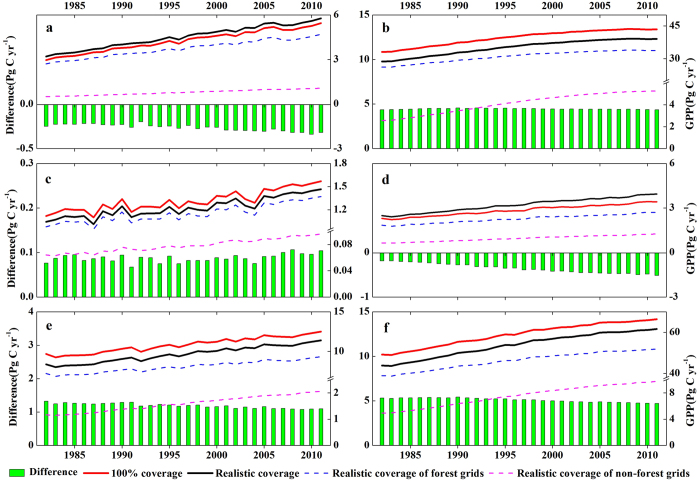
Interannual variation in GPP (Pg C yr^−1^) for different forest types during 1982−2011. **a**, ENF. **b**, EBF. **c**, DNF. **d**, DBF. **e**, MIF. **f**, forest ecosystems. The figure was produced using OriginPro 8.1.

Forest area, another critical factor for globally annual GPP, has not been paid enough attention in the previous studies. Here, we argue land cover classification and coverage rate are two major causes for the different estimates in global forest area. For example, a previous study has revealed changes in land cover maps could result in large differences in global GPP estimates^1^. In the present study, EBF exists in south-central Africa (e.g., Afghanistan, Zambia, Mozambique, and Southern Congo) based on IGBP-DIS classification (Fig. 1a); however, according to global land cover 2000 (GLC2000) and moderate resolution imaging spectroradiometer (MODIS) land cover, the identical land is broadly classified into DBF and savanna, respectively. Hence, it is evident that inconsistent forest area of land cover classification maps could directly increase the uncertainty of globally annual GPP in forest ecosystems. With respect to coverage rate, most previous studies assumed 100% coverage of each spatial grid by a given forest type, whereas the IGBP-DIS classification shows that the coverage rate along the periphery of the forest is only 40−70% (Fig. 1b), and the averaged value of each grid is approximately 80.6% (Fig. 4), which largely deviates from the assumption of 100% coverage; meanwhile, some land cover grids not categorized as forest also contain a certain amount of forest area (Fig. 1c) and the averaged coverage rate could nearly approach to 3.5% (Fig. 4), which is absolutely not a negligible factor for estimating forest carbon fluxes on large scale. For these reasons, both the neglected forest coverage of forest grids and non-forest grids by previous studies could indeed overestimate and underestimate forest area, respectively, and therefore, we argue that their conclusions concerning carbon dioxide uptake by global forest ecosystems are likely to be further discussed and reevaluated. Besides, the contributions of land use and land cover change (LULCC) to anthropogenic carbon emissions are approximately 12.5% over the 2000−2009 period^18^, and the largest carbon emissions by LULCC have been from land cove change, particularly the conversion of forests to non-forests, or deforestation^19^. Here, we also infer that the satellite-based approach used to document changes in forest area would increase the uncertainty of carbon emissions from LULCC, if the coverage rate is not properly considered in the estimation process.

In our study, the overestimated GPP in global forest ecosystems caused by coverage rate is approximately 5.12 ± 0.23 Pg C yr^−1^. This amount is equivalent to 48% of the annual emissions from anthropogenic activities in 2012 (9.7 ± 0.5 Pg C yr^−1^ for fossil-fuel combustion and cement production and 1.0 ± 0.5 Pg C yr^−1^ for land-use change)^20^. Despite this conclusion, to some extent, large uncertainty remains and requires further discussion, particularly regarding the accuracy of coverage rate in the IGBP-DIS classification. We argue that coverage rate still may be a required factor to further quantify the global carbon cycle.

## Methods

### FORCCHN model

The model used in the study is FORCCHN, which is constructed to simulate seasonal and interannual variations in the carbon budget of forest ecosystems in the given areas^21^. FORCCHN model is built on two different timescales, including daily and annual processes. Primary daily processes are composed of photosynthesis, plant respiration, photosynthate allocation, litter production, and soil respiration and transfer, while annual processes consist of distributions of forest stand assimilation, growth in the form of tree height and diameter at breast height (DBH), and the production of coarse wood debris(CWD). Detailed description of the daily processes and relevant computations concerning the increment of wood, basal diameter and tree height can be found in refs 21 and 22.

In FORCCHN, the daily GPP of an individual tree (kg C d^−1^) is given by:
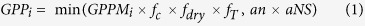

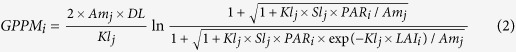
where *GPPM*_*i*_ is the maximal daily gross primary production of the ith tree (kg C d^−1^); *f*_*c*_*, f*_*dry*_*, f*_*T*_ and *an × aNS* represent the effects of carbon dioxide, water, temperature and soil active nitrogen on GPP, respectively; *aNS* is the soil active nitrogen amount (kg N m^−2^) and *an* = 150; *DL* is the possible sunshine duration (h); *PAR*_*i*_ is the photosynthetic active radiation of the canopy at noon (W m^−2^); *LAI*_*i*_ is the leaf area index of the ith tree; *Am*_*j*_, *Kl*_*j*_ and *Sl*_*j*_ represent the maximal photosynthesis (kg C m^−2^ h^−1^), the extinction coefficient and the initial slope of light intension and photosynthesis (kg C W^−2^ h^−1^) of the jth forest type, respectively. 

### Model forcing data on the global scale

FORCCHN is driven by climatic conditions, soil parameters and forest characteristics. **I.** Climatic conditions consist of the daily maximum and minimum air temperature (°C), precipitation (mm), relative humidity (%), wind speed (m/s), atmospheric pressure (hPa) and total solar radiation (W m^−2^).In this paper, we used the global meteorological forcing dataset from Princeton University (PU dataset) over the period of 1982−2011 at a grid resolution of 0.5° × 0.5°^23^.This dataset provides near-surface meteorological data for driving land surface models and other terrestrial modeling systems. It blends reanalysis data with observations and disaggregates in time and space. **II.** Soil parameters are composed of the soil organic matter (carbon and nitrogen pool in units of kg C m^−2^ and kg N m^−2^, respectively), soil physical parameters and litter pool decomposition parameters. The soil physical parameters, which are strongly dependent on the geographical position, include the soil field capacity (mm), wilting point (mm), bulk density (kg m^−3^), sand content (%), silt content (%) and clay content (%).In the present study, the Global Gridded Surfaces of Selected Soil Characteristics^24^, coupled with the Harmonized World Soil Database^25^, provides resources for the soil organic matter and physical parameters. Furthermore, the litter pool decomposition parameters can be found in refs 21 and 22. **III.** We ascertained the global forest types (Fig. 1a), forest coverage rate of forest grids (Fig. 1b) and non-forest grids (Fig. 1c) based on International Geosphere-Biosphere Program-Data and Information Service (IGBP-DIS) *DISCover* land cover classification system with a spatial resolution of 0.5° × 0.5°^26^. In addition, the 8-day 5-km LAI of global land surface satellite (GLASS) in 1982^27^ is also used to drive the model. Quality control flags in LAI were examined to screen and reject poor quality data. The 8-day GLASS data were composited into yearly maximum and minimum values. Note that the satellite datasets were resampled to the geographic projection and spatial resolution of the global PU dataset.

### GPP estimation in Calculation II

On the basis of IGBP-DIS classification, a given land cover grid might include several forest types irrespective of whether the grid is forest or non-forest (Fig. 4). Accordingly, in order to estimate the total forest GPP(Tg C yr^−1^) at each grid, we firstly simulate the GPP per unit area (g C m^−2^ yr^−1^) of included forest types using FORCCHN model, and then multiply by the gird area (m^2^) and corresponding coverage rate (%) to get their total GPP, respectively. Finally, these kinds of total GPP for different forest types are added up and we take the aggregated GPP as the total forest GPP at each grid. The equation is list as follows:
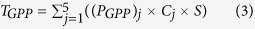
where *T*_*GPP*_ is the total forest GPP (Tg C yr^-1^) at each grid; *j* is an integer variable, delineating 5 forest types and ranging from 1 to 5; (*P*_*GPP*_)_*j*_ and *C*_*j*_ represent the GPP per unit area (g C m^−2^ yr^−1^) and the coverage rate (%) of the jth forest type, respectively; *S* is the corresponding grid area (m^2^).

## Additional Information

**How to cite this article**: Ma, J. *et al.* Gross primary production of global forest ecosystems has been overestimated. *Sci. Rep.* **5**, 10820; doi: 10.1038/srep10820 (2015).

## Figures and Tables

**Table 1:**
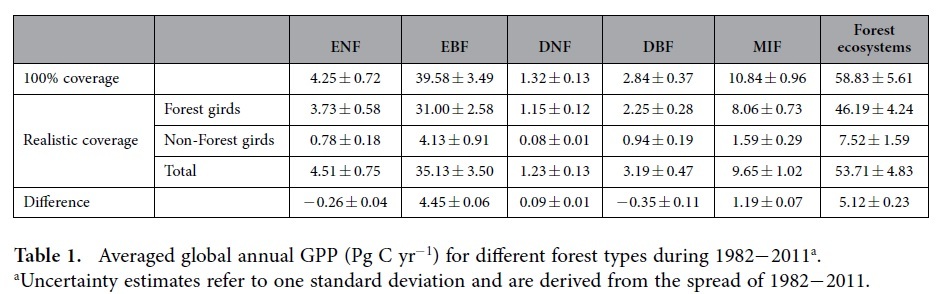
Averaged global annual GPP (Pg C yr^−1^) for different forest types during 1982−2011^a^.
